# The Warburg Effect Explained: Integration of Enhanced Glycolysis with Heterogeneous Mitochondria to Promote Cancer Cell Proliferation

**DOI:** 10.3390/ijms242115787

**Published:** 2023-10-31

**Authors:** Lilia Alberghina

**Affiliations:** Centre of Systems Biology, University of Milano-Bicocca, Piazza della Scienza 2, 20126 Milan, Italy; lilia.alberghina@unimib.it

**Keywords:** cancer, mitochondria, OXPHOS, ATP synthase, ROS, cellular biochemistry

## Abstract

The Warburg effect is the long-standing riddle of cancer biology. How does aerobic glycolysis, inefficient in producing ATP, confer a growth advantage to cancer cells? A new evaluation of a large set of literature findings covering the Warburg effect and its yeast counterpart, the Crabtree effect, led to an innovative working hypothesis presented here. It holds that enhanced glycolysis partially inactivates oxidative phosphorylation to induce functional rewiring of a set of TCA cycle enzymes to generate new non-canonical metabolic pathways that sustain faster growth rates. The hypothesis has been structured by constructing two metabolic maps, one for cancer metabolism and the other for the yeast Crabtree effect. New lines of investigation, suggested by these maps, are discussed as instrumental in leading toward a better understanding of cancer biology in order to allow the development of more efficient metabolism-targeted anticancer drugs.

## 1. Introduction

Next year, in 2024, the discovery of the Warburg effect [1] will celebrate its 100th anniversary. Although it has been foretold that “no one can doubt that we understand the origin of cancer cells if …. we know how the damaged respiration and the excessive fermentation of the cancer cells originate” [2], no satisfactory biochemical mechanism has so far been put forward to account for the peculiar metabolism of cancer cells, which appears to contradict the widely held idea on the inefficiency of fermentation in supporting the production of biomass. After the long period during which the genetic origin of cancer has been considered the only field worth of attention, the last 15 years or so have seen a profound reappraisal of the role of nutrient utilization, including glutamine [3,4], in connection with respiro-fermentative metabolism, to generate cancer proliferation, the more so since the Warburg effect is so reliable to be used for medical diagnosis of tumors, utilizing fluoro-deoxy glucose positron-emission tomography (FDG-PET) [5]. The Warburg effect is not a peculiar feature of cancer cells: it is one aspect of a profound yet unknown regulatory mechanism of metabolism detected in organisms with different evolutionary complexity, such as yeast, where it is called the Crabtree effect [6]. It would be possible to take advantage of the versatility of yeast genetic and biochemical approaches to deal with the complexity of metabolic regulation in yeast and cancer cells [7]. 

Disentangling the dynamics of the main metabolic pathways active in yeast or cancer cells will allow us to reconstruct the maps of the metabolic pathways for both organisms. These maps will help to understand the regulatory mechanism that determines both the Warburg and the Crabtree effects and identify the more relevant steps controlling the rate of cell proliferation. Given the availability of a wealth of literature reports on both effects, this paper has surveyed a large set of published experimental findings allowing recognition of the “fil rouge”, which permits an enhanced, apparently energetically inefficient fermentation and damaged respiration to support increased growth rate. 

The present investigation is necessitated by the observation that, while the Warburg effect is generally detected in most different tumors, the metabolic vulnerability to drugs has been reported to be largely variable in different cancer cells and able to undergo unpredictable evolution during tumor progression or after drug treatments [8,9]. Given that the advancements of technologies (multi-omics, single cell, and spatial analyses) may allow to dynamically and spatially characterize cancer cell metabolism, it would be extremely useful to have a better understanding of the insurgence of variable metabolic vulnerability and its possible, dynamic connection with the Warburg effect, which continues to be taken as a relevant hallmark of cancer. 

The breakthrough hypothesis presented in this report proposes that enhanced glycolysis induces a rewiring in the synthesis and in the functional assembly of mitochondrial TCA cycle enzymes in mammalian (cancer) and in yeast cells undergoing the Warburg and Crabtree effects to generate mitochondrial heterogeneity different in yeast and in cancer cells and new metabolic pathways able to sustain increased cell proliferation. 

This new perspective is very important because it may offer solid ground to design and realize new therapies targeting cancer metabolism and to improve their clinical efficacy [10,11,12,13].

## 2. The Crabtree Effect: A New Map of Its Integrated Metabolic Network

Not all yeast species undergo the Crabtree transition. Only yeasts that, in high-glucose media, can utilize large amounts of glucose may activate the Crabtree transition; they are called Crabtree-positive yeasts [14]. Crabtree-positive yeasts, such as Saccharomyces cerevisiae, undergo, as previously anticipated, a metabolic shift from a fully respiratory metabolism in low glucose (or in a poor carbon source, such as ethanol or glycerol) to a respiro-fermentative metabolism in high glucose. The Crabtree transition lasts 7–8 h at 30 °C [15]. During the transition period, growth and cell cycle progression are strongly affected: cells are blocked at the G1/S checkpoint for a significant period of time; ribosomal RNA content and rate of protein synthesis are enhanced; newborn daughter cells increase in size [15]. Additionally, an extensive reorganization of protein set-up takes place: protein allocation to various function units (protein synthesis, glycolysis, mitochondria, lipid metabolism) changes from respiratory to respiro-fermentative metabolism [16]. Finally, Crabtree-positive yeast cells in high glucose have a much reduced OXPHOS electron transfer chain activity and a much lower ATP production [14,15,16,17]. In summary, Crabtree-positive yeasts, when grown in low glucose, present a respiratory metabolism and low growth rate; increasing glucose availability, enhances the growth rate, maintaining a high yield (amount of biomass produced per unit of utilized glucose). After reaching a critical value of glucose availability, the Crabtree effect becomes apparent: cells shift to a respiro-fermentative metabolism, which supports further increased growth rate (and protein synthesis rate), but a reduction in the yield, and finally, stimulated production of ethanol [18]. 

Given that budding yeast is extensively utilized in biotechnological processes for the production of chemicals, biofuels, and therapeutic proteins, the occurrence of the Crabtree effect strongly reduces the productivity of these industrial processes. Many studies have been carried out to increase the productivity of yeast biotechnological processes, trying to overcome the Crabtree effect so to obtain high cellular yields at fast growth rates in continuous cultures. It has been shown that additions of fumaric acid and malic acid are able to reduce ethanol production in continuous cultures at high dilution rates [19], confirming a pattern observed with the addition of formate [20], suggesting that a stimulated production of NADH may reduce ethanol formation. Comparison of metabolic profiles of yeast Crabtree positive and negative yeasts indicates a relevant role of NAD+/NADH in establishing the Crabtree effect [21]. A more detailed analysis indicates that the Crabtree effect is achieved by a global rewiring of metabolism, which may be experimentally accomplished by disruption of the pyruvate decarboxylase gene followed by an adaptive laboratory evolution, able to generate a mutation of the Mediator complex of RNA polymerase II [22,23].

The global rewiring of metabolism generated by the Crabtree effect has been investigated by multi-omics analysis, which detected a determinant activation of the citrate/malate and the oxaloacetate/H^+^ shuttles [24]. The citrate and oxalacetate imported by these shuttles in the mitochondria promote carbon flux preferentially towards two sections of the TCA cycle:

citrate → isocitrate → α-ketoglutarate    (clockwise) NAD^+^ → NADH (1)

oxaloacetate → malate    (counterclockwise) NADH → NAD^+^ (2)

The cytoplasmic pathway: pyruvate → oxaloacetate → citrate is also upregulated, while the TCA cycle reactions: α-ketoglutarate → succinate → fumarate → malate, are downregulated due to the reduced entrance of Acetyl-CoA into the TCA cycle [24].

These findings made me wonder how reactions of the canonical TCA cycle may respond in different ways during the Crabtree transition, possibly indicating the activation of a new pathway. Reactions (1) and (2) show an enhanced flow of carbon, and the result is redox-balanced since reaction (1) transforms NAD^+^ into NADH, while reaction (2) converts NADH into NAD+. Thus, they may continue to proceed as a redox-powered cycle, even when the electron flow going to the OXPHOS chain is drastically reduced, a condition occurring during the Crabtree effect [14]. These findings suggest a relevant yet unexpected role of differential mitochondrial enzyme synthesis and possibly also of a different supramolecular enzyme assembly during the development of cellular responses in the Crabtree effect. 

Why is the OXPHOS electron transfer chain strongly reduced during the Crabtree effect? Flux balance analysis, combined with “in vitro” determination of enzyme-specific activity, has shown that fermentation produces more ATP per protein mass unit than mitochondrial respiration [25]. So, in terms of growth efficiency, there is a trade-off between fermentation and respiration. The enzyme F1F0-ATP synthase has been shown to have flux control over respiration [25]. It follows that, in order to realize the Crabtree effect, the F1F0-ATP synthase should be inhibited. Due to the evolutionary conservation of F1F0-ATP synthase, it is expected that this trade-off will be active both in yeast and in humans [25]. 

**Figure 1:**
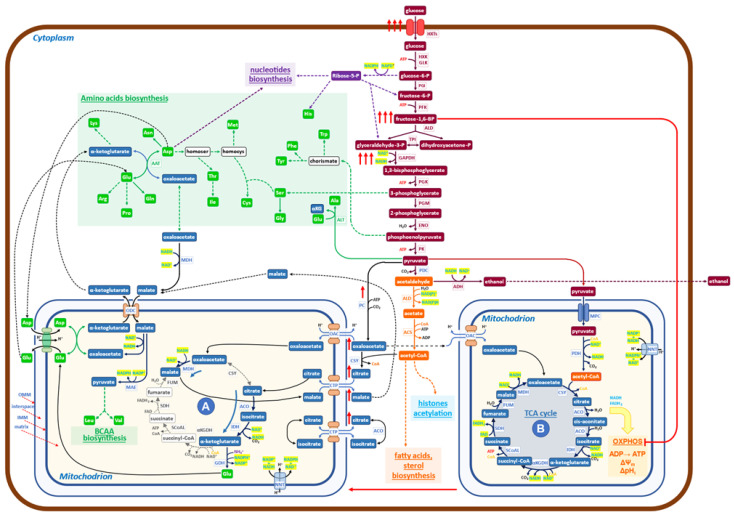
Schematic diagram of the metabolic network governing the Crabtree effect in yeast. At high glucose availability, Crabtree-positive yeasts respond by increasing the uptake of glucose (red arrows) and the glycolytic flux. Hence, the level of Fructose 1,6 BP is increased (red arrows) to inhibit the OXPHOS functionality (red line) to facilitate the formation of a new mitochondrial functionality (A), which carries on a downsized, non-canonical TCA cycle, devoid of all bioenergetics functions (from OXPHOS electron transfer to membrane potential). The increased glycolytic flux produces large amounts of NADH at the level of 3-phospho-glyceraldehyde dehydrogenase, which needs to be re-oxidized to allow the enhanced glycolysis to proceed. The malate dehydrogenase (MDH) may utilize NADH to produce malate from oxaloacetate in the cytoplasm. The malate/aspartate shuttle transfers malate into mitochondria, where it is transformed into oxaloacetate, releasing NADH. In mitochondria (A), the newly formed NADH stimulates the reduction of oxaloacetate to malate, receiving oxaloacetate from one of the shuttles, refilled by pyruvate, coming from the enhanced glycolysis (red arrows). Additionally, citrate is imported and transformed to isocitrate and then to α-ketoglutarate, which, by reductive transamination, powered by NADPH, becomes glutamate, to be exported in the cytoplasm. In conclusion, the role of mitochondria (A) is to receive products derived from pyruvate and to transform them into glutamate, α-ketoglutarate, and malate, which may generate, together with products coming from glycolysis, all the building blocks necessary to produce new biomass (nucleotides, proteins, fatty acids, etc.). It has been shown that growth and protein synthesis are significantly stimulated during the Crabtree transition (9). Mitochondria A may allow faster building blocks production than canonical mitochondria (B) since its reactions are faster than the similar ones carried out by mitochondria (B), which are coupled to slow reactions of electron transfer in OXPHOS and ATP synthesis by ATP synthase. The utilization of pyruvate by the two mitochondrial structures, (A) and (B), does not match the production of pyruvate coming from enhanced glycolysis, so a sizable aliquot goes to ethanol, allowing both to balance the redox state of NADH/NAD+ and to increase the amount of ATP, made at the level of glycolysis, for cellular works.

It has been reported [26] that the highly stimulated glycolysis increases the production rate and the level of fructose 1,6-bisphosphate (FBP) (Figure 1), which has been found to inhibit mitochondrial oxidation activity, with a significant reduction in cytochrome-C oxidase activity, as assayed “in vitro” on permeabilized spheroplasts in Crabtree-positive yeast. The FBP-mediated inhibition was also observed in isolated rat liver mitochondria, extending this regulatory event to mammalian cells [26]. Further studies indicate a more complex regulatory pattern: the electron flux in the mitochondrial respiratory chain is inhibited by FBP, but it is activated by another glycolytic intermediate, glucose-6-phosphate (G6P). Only when the concentration ratio FBP/G6P surpasses one, the induction of the Crabtree effect will take place [27]. Genetic and nutritional manipulations may affect the FBP/G6P ratio and hence the onset of the Crabtree effect [27].

It has to be noticed that “in vitro” assays, at increasing concentrations of FBP, at most a 50% inhibition of cytochrome-C oxidase activity is obtained, indicating that, even at fairly high cytoplasmic concentrations of FBP, it is likely that two types of mitochondrial activities may coexist: one able to carry on canonical TCA cycle with active OXPHOS activities and a second one, characterized by OXPHOS inhibition, which may carry on only the previously described redox-powered cycle. Given that it is difficult to hold that carbon flux may proceed with different rates and orientations on an individual TCA cycle, it seems reasonable to hypothesize that two “types” of mitochondria may be present in the Crabtree effect. The first type should carry on a downsized non-canonical TCA cycle, in which only reactions (1) and (2), would be active (Figure 1 (A)). The second type carries on the canonical TCA cycle, electron transfer chain flux on OXPHOS, and oxidative phosphorylation (Figure 1 (B)). 

In conclusion, the rise of FBP in the cytoplasm, as well as the inactivation of F1F0-ATP synthase during the Crabtree transition, would partially inhibit OXPHOS functionality, producing a set of mitochondria (or of mitochondrial cristae in otherwise normal mitochondria), able to carry on downsized non-canonical TCA cycle. As already reported [24], this non-canonical TCA cycle needs to receive substrate feeding from the cytoplasm and export products of its reactions into the cytoplasm. A different type of non-canonical TCA cycle has been reported in the literature for mammalian cells, and it will be discussed later on.

To better appreciate the biological significance of the findings collected so far on the metabolism characteristic of the Crabtree effect, let us comment on Figure 1, in which the new findings on metabolism activated during the Crabtree transition have been inserted into a basic biochemical map. Under the Crabtree effect, glycolysis is strongly stimulated so that NADH, produced at the level of 3-phospho-glyceraldehyde oxidation, needs to be quickly re-oxidized to allow the glycolytic flux to proceed at a fast rate. In eukaryotes, such as yeast, the malate/aspartate shuttle can transfer reducing equivalents of NADH from the cytoplasm to the mitochondrial matrix [28,29]. The increase in NADH concentration in mitochondria (A) stimulates, for thermodynamic reasons, the conversion of oxaloacetate to malate (Figure 1). The ensuing production of NAD+ will promote the formation, from isocitrate, of α-ketoglutarate. This latter compound may then be converted to glutamate [24] by using NADPH, derived from NADH by the action of NNT (nicotinamide nucleotide transhydrogenase). Glutamate is exported into the cytoplasm by the glutamate/malate shuttle, which is formed by two coupled antiporters (glutamate/aspartate and α-ketoglutarate/malate), which have the main function of conveying in mitochondria the excess of NADH formed in the cytoplasm to be used for biosynthetic purposes [30].

The enhanced malate production in mitochondria (A) stimulates the entrance of citrate (in exchange in the citrate/malate shuttle), allowing a new round of redox-balanced reactions to proceed, sustained by the upstream enhanced glycolytic flux. 

In summary, mitochondria (A) receive from the cytoplasm: oxaloacetate, citrate, malate, and aspartate; and export: α-ketoglutarate, glutamate, and malate (Figure 1). While the first two products are going to generate, together with substrates coming from glycolysis (including Acetyl-CoA), the building blocks required for growth (amino acids, nucleotides, fatty acids), malate appears to have the role not only to carry electrons from cytoplasmic NADH into mitochondria (A) but also to activate a positive feed-back to promote the activity of the downsized non-canonical TCA cycle (Figure 1). Of course, further investigations will allow us to complete the set of biochemical reactions taking place in mitochondria (A), besides those reported in Figure 1, the only ones for which, so far, experimental evidence is available [24].

A reason why the downsized non-canonical TCA cycle may offer a pathway able to sustain a faster rate of protein synthesis (and hence of growth) in comparison with canonical TCA cycles may be due to the fact that the canonical TCA cycle is rate-limited by the transfer of electrons to the OXPHOS chain and by the conversion of electrochemical potential into ATP synthesis. Hence, mitochondria containing the complete enzyme set of the canonical TCA cycle indicated as (B) in Figure 1, are responsible for the Krebs cycle reactions, OXPHOS electron transfer, oxidative phosphorylation, generation of membrane potential, and pH control. The inhibitory effect of FBP and F1F0-ATP synthase downregulation is expected to partially convert mitochondria (B) into mitochondria (A) [26,27]. 

In conclusion, the molecular engine’s ability to sustain faster growth during the Crabtree transition is taken to be due to the activation of the non-canonical downsized TCA cycle, promoted by enhanced glycolytic flux. In terms of systems biology [31], we may consider the Crabtree effect as the “emergent property ” of the complex network of metabolic reactions, sketched in Figure 1.

## 3. The Warburg Effect: A New Map of Its Integrated, Metabolic Network

The neoplastic transformation has been recognized to involve many cellular functions and molecular pathways [32]. However, it has been impossible so far to identify which event is directly responsible for the deregulated, stimulated growth ability of cancer cells, which is the crucial phenotype linked to the disease. 

Metabolism produces building blocks for growth, so it is likely that a metabolism remodeling may take place to become able to support the enhanced requirement of biochemical compounds to be used for biomass formation. The Warburg effect, in which aerobic fermentation correlates with a stimulated proliferation rate, is a promising candidate for the search for critical regulatory functions of cancer. The insertion of deregulated cellular energetics (covering from glycolysis to oxidative phosphorylation) in the hallmarks of cancer [32] and the discovery of a strong dependence of cancer cell growth from the availability of glutamine (3,4) in the medium are further indications in favor of this hypothesis. 

A significant co-regulation of enhanced glycolysis and glutamine utilization has been reported in cancer cells. For instance, the MYC gene positively controls both the expression of the glycolytic enzymes, glutamine uptake, and glutaminase activity [33,34]. Glutamine deficiency induces MYC-dependent apoptosis in cancer cells [35] and abortive S-phase arrest in K-ras transformed cells [36]. 

Glutamine provided to cancer cells is initially converted to glutamate, which is transaminated to α-ketoglutarate, then transformed by reductive carboxylation, to isocitrate and finally, by aconitase, to citrate [37,38]. The reductive carboxylation reaction is a marker of cancer cells, being carried on by mutants of isocitrate dehydrogenase [39]. Isocitrate dehydrogenase in humans is encoded chiefly by two genes, IDH1 and IDH2, which express different isocitrate dehydrogenases, dependent upon either NADPH or NADH, which localize in the mitochondrial matrix or in the cytosol. The reductive carboxylation of α-ketoglutarate may utilize NADH, but if NADPH is required, the NNT enzyme (nicotinamide nucleotide transhydrogenase) [40], localized on the inner membrane of mitochondria, may interconvert NADH and NADPH.

**Figure 2:**
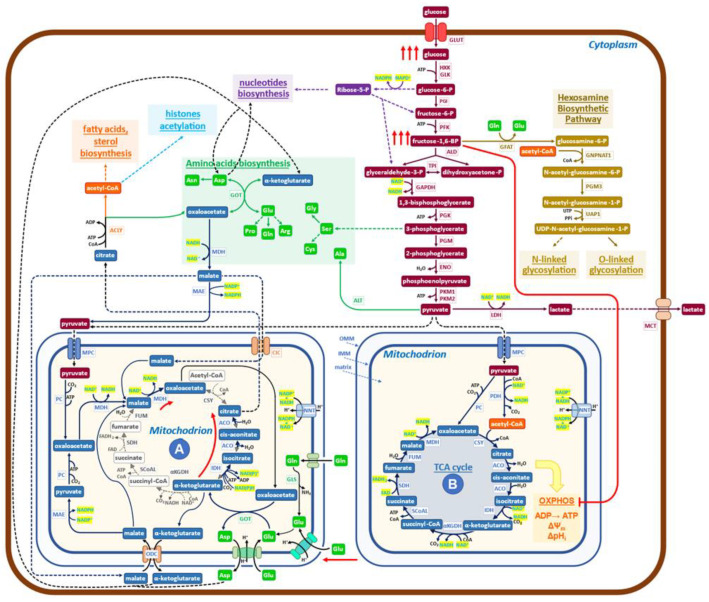
Schematic diagram of the metabolic network governing the Warburg effect in cancer cells. The glucose uptake rate of cancer cells is quite high (red arrows), and enhanced is also the production of lactate. The increased level of FBP inactivates (red line) the OXPHOS pathway and ATP production to generate the indicated supramolecular assembly of mitochondrial enzymes (red arrows), devoid of bioenergetics activities. Mitochondria (A) of cancer cells formed in this way utilize glutamine through reductive carboxylation to generate citrate, which is exported into the cytoplasm by the malate/citrate shuttle. Pyruvate is imported into mitochondria to promote its conversion to oxaloacetate, producing NADH, which stimulates the rate of reductive carboxylation of α-ketoglutarate and the export of citrate into the cytoplasm. Oxaloacetate collaborates to increase the production of citrate by furnishing, through transamination, new α-ketoglutarate. Pyruvate, coming from glycolysis, enters mitochondria (A) to generate new NADH, which, by the activity of NNT (nicotinamide nucleotide transhydrogenase), may generate NADPH, able, for chemical kinetics, to stimulate the production of citrate. A malate/aspartate shuttle exports aspartate and α-ketoglutarate into the cytoplasm while simultaneously participating in glutamate metabolism. In conclusion, the biochemical activities of mitochondria (A) utilize glutamine, glutamate, malate, and pyruvate and export citrate, α-ketoglutarate, and aspartate into the cytoplasm. A relevant role is taken by cytoplasmic ACLY (ATP citrate lyase) in controlling the pathway that generates building blocks for growth.

In summary, the characteristic pathway of glutamine utilization in cancer [41], which is clearly a counterclockwise reaction of the canonical TCA cycle, may be outlined as follows (Figure 2):

glutamate → α-ketoglutarate → isocitrate → citrate   (counterclockwise) NADH → NAD^+^ (3)

The aspartate/glutamate shuttle [30], which is engaged in glutamate utilization by the non-canonical TCA cycle, introduces malate into the mitochondrial matrix, bringing to the activation of the reaction (4):

malate → oxaloacetate     (clockwise) NAD^+^ → NADH (4)

which redox balances the reaction (3).

Reactions (3) and (4) are the same that have been detected in the Crabtree effect, but they flow in opposite directions, thereby indicating that there are only these two solutions to generate redox-sustained non-canonical, downsized TCA cycles. The pivotal role played by glycolytic intermediates, FBP and G6P, in promoting the onset of the Crabtree effect has been previously reported [26,27]. More precisely, only when the concentration ratio FBP/G6P exceeds one will the onset of the Crabtree effect take place [27]. Findings recently obtained by determining the metabolic abundance profiles in normal human colon CCD-18Co cells and in HCT 15 colon cancer cells [42] support the notion that the regulatory design, triggered by FBP/G6P, may also be extended to the Warburg effect. In fact, it is reported that in cancer cells, the level of FBP is much higher than that of G6P, while in normal cells, the level of G6P is substantially larger than that of FBP [42]. Further, it has to be recalled that the FBP-mediated inhibition of mitochondrial cytochrome-C oxidase activity has been reported to occur also in mammalian cells (isolated rat liver mitochondria) [26].

The data so far collected on cancer cell metabolism, as well as the diffuse opinion, of which [43] is an example, of the evolutionary conservation of the Crabtree and Warburg effects, supports the notion that the two metabolic effects are so similar that it would be conceivable to use the map, reported in Figure 1 for the Crabtree effect, as the mold for the new map, that will aim to describe the more relevant metabolic reactions that in cancer cells generate the Warburg effect (Figure 2). 

Let us now verify, for Figure 2, whether two relevant features reported in Figure 1 network for the Crabtree effect are validated in cancer cells. An increased uptake of glucose, stimulated by growth factors mostly oncogene-dependent, is detected in cancer cells [44]; the inhibition of mitochondrial oxidative phosphorylation takes place in cancer cells both due to the increase in FBP concentration (24, thereby generating mitochondrial structures devoid of OXPHOS activity, but still able to carry on redox-balanced reactions in a variation of the TCA cycle. Recent reports have shed new light on the functional heterogeneity of mitochondria. Imaging analysis has shown that mitochondrial cristae display different membrane potentials within the same mitochondrion, implying differences in OXPHOS electron transfer chain activity and oxidative phosphorylation [45,46]. Not only that, in glioblastoma multiforme mitochondria, it has been reported that the dissolution of cristae is indicative of loss of OXPHOS activity [47]. Further, the ATP production in the cytosol is linked to the morphology of mitochondria [48].

More informative on the question of mitochondrial heterogeneity in mammalian cells is the paper from Arnold et al. [49], which reveals, by genetic co-essentiality mapping, the existence of a cluster of genes that appears to be able to generate a biochemical pathway, alternative to the canonical TCA cycle. In this new pathway, citrate is exported into the cytoplasm by the citrate/malate shuttle; then it is converted by ATP citrate lyase (ACLY) to oxaloacetate, which has been proposed [49] to re-enter the TCA cycle. This last interpretation does not appear to be in accord with the observation that inhibition of ACLY causes potent growth inhibition [50]. On the other hand, the authors of [49] sustain that the citrate/malate shuttle should have a relevant role in cell metabolism, while the function they propose is just to refuel oxaloacetate to mitochondria. The findings reported in Figure 2 of the cited paper [49] may also be interpreted in the frame of the pathways presented in Fig 2 of the present report, both for the labeling profiles and for the observed inhibitory effects due to ACLY inactivation on growth. In embryonic stem cells, inhibition of the non-canonical TCA cycle blocks the exit from pluripotency, preventing the embryo’s normal development, which needs increased and diversified protein synthetic activity [49]. These findings support the notion that the non-canonical TCA cycle of [49] may be better explained by the map of Figure 2.

Another indication that the TCA cycle does not always follow the simple hard-wired circular pathway, that biochemistry books and thousands of scientific papers have impressed in our minds for decades, comes from another recent paper [51]. The glycolysis and TCA cycle rates in healthy tissues and tumors have been measured in mouse models “in vivo”. Tumors make ATP more slowly than healthy tissues, while metastases have a TCA cycle rate faster than orthotopic tumors. Given that tumors grow and show cell division, cancer cells need to maintain the ability to produce building blocks for growth, even if their ATP production is low. On the other hand, healthy tissues showing a high ATP production rate are known to carry on substantial cellular works besides growth biosynthesis (by way of example, contraction in the heart and diaphragm and filtration in the kidney) [51]. These findings agree with the well-known reduced activity of the OXPHOS electron transfer chain in cancer cells, either due to mutations in the OXPHOS chain components [52] or to inhibition of ATP synthase activity [53,54,55,56,57]. 

Taken together, these reports suggest that a downsized, non-canonical TCA cycle is active in cancer cells and in mammalian cells in the early stages of embryogenesis, as shown in Figure 2, in which the findings described above have been inserted in a standard, simplified map of cancer cells metabolism. Characteristic is the presence of mitochondria (A) in which the downsized non-canonical TCA cycle is shown. Mitochondria (A) receive both glutamate and pyruvate from the enhanced glycolysis. Following reductive carboxylation, they produce citrate, exported into the cytoplasm by the citrate/malate shuttle [53,54,55,56,57,58], to be converted by ACLY into oxaloacetate and acetyl-CoA. Then, an extensive network of reactions will generate the building blocks required for growth (synthesis of protein, lipids, and nucleic acids, which utilizes both substrates coming from glycolysis and oxaloacetate, α-ketoglutarate and aspartate coming from mitochondria), using the redox potential of NAD(P)H produced by malate dehydrogenase (MDH) and malic enzyme (ME1) (Figure 2). It may be worth recalling, at this point, that the presence of formate in the medium promotes invasion and metastasis in cancer cells, requiring a functional lipid metabolism to activate the pro-invasive activity [59].

The biochemical network, which sustains the Warburg effect (Figure 2), follows a design similar to that presented before for the Crabtree effect but with clearly different arrangements for several players. Reactions (1) and (2) in yeast have one orientation, while the same reactions, which become (3) and (4) in cancer cells, follow the opposite directions (see Figure 1 (A) and Figure 2 (A)). Hence, the input and output molecules substantially differ between the two effects. 

Given that there are only these two ways to generate downsized non-canonical TCA cycles, one may wonder whether a variant of the network, described herein for the Crabtree effect (Figure 1), may be active also in cancer cells, especially in those cells that have inhibited glutamine utilization, but are still able to proliferate. 

So far, only a restricted number of shuttles has been considered (Figure 2), while it is known that the number of mitochondria transporters involved in different cancer types is much larger [60]. It remains to be investigated the possibility that in some tumors, different sets of shuttles may generate variants of the two basic patterns of non-canonical downsized TCA cycles, uncovered in this note.

Finally, it would be of interest if a systems biology approach [61] may allow the development of dynamic mathematic models able to yield an understanding of features of cancer metabolism yet unexplained. Why does PKM2 [62] delay glycolytic flux? It is to coordinate the rates of production of building blocks for growth, which are going to have, as precursors, both molecules coming from glycolysis and from the non-canonical TCA cycle? Another question to be investigated is the role of the saturation of NADH shuttle in the partition of pyruvate between entrance into the canonical TCA cycle or dehydrogenation to lactate [63] or, finally, supports the non-canonical TCA cycle (Figure 2 (A)). A better appreciation of the molecular networks in cancer than that presently available [64] may be derived.

Alterations of metabolism have been recognized as a hallmark of cancer cells [65], and the utilization of metabolic models has been proposed as a possible relevant tool for precision medicine [66,67,68]. New interesting perspectives are added by the present report.

## 4. Complexity of Cancer Metabolism

The large differences observed when comparing transcriptome profiles between healthy and cancer cells or among various cancer types have encouraged the search for prognostic gene signatures, which would be useful for the development of improved strategies for the treatment of patients [68,69,70]. 

To better appreciate the comparison of metabolic profiles, one must consider the complexity of overall metabolism and remember that “in vivo”, the tumor microenvironment (TME) dynamically interacts with cancer cells so that data collected on cancer cell lines grown as standard two-dimensional cultures may provide an over-simplified view of the metabolism of the whole tumor.

The human metabolic network accommodates a huge number of molecules: the more recent estimate, made using the Recon 3D model [71], reports: 3288 open reading frames (that is 17% of the functionally annotated human genes), 13,543 metabolic reactions, which engage 4140 metabolites. Metabolism may be divided into: central carbon metabolism, biosynthesis, and secondary metabolism. The three areas of metabolism are dynamically interlinked, especially by connections given by common utilization of energy molecules (ATP, ADP, etc.) and of redox ones (NADH/NAD^+^; NADPH/NADP^+^), which are deeply involved in reactions of central carbon metabolism, while at the same time, they impact on rate and orientation of tens, hundreds of reactions of the other two sections. It follows that the redox state, as well as the energy charge, are largely responsible for the dynamics of fluxes on the entire network of expressed metabolic enzymes. Typically, the carbon flux in the first section (the central carbon metabolism) is larger by a factor of 10 or more [72] compared to the other two sections, thus giving central carbon metabolism a significant weight on the overall metabolic dynamics. Not only, the secondary metabolism, which often constitutes the larger section of the metabolic network, is in prevalence determined by the differentiation fate of each cell, so that identical stimuli, able to induce specific alterations (for instance, the activation of a signaling pathway by an oncogene), may impact, in significantly different ways, on metabolic fluxes of different cells and organs [73] and hence on their transcriptomics profiles. 

The comparison of genome-scale metabolic models of 917 primary tumor samples of 13 different types conducted by using transcriptome data indicate that the metabolic networks in different cancers are largely accounted for using the same reactions despite limited differences in the expression level of the transcripts encoding individual metabolic enzymes. Only a few scattered genes are found to be differentially expressed in some tumor types [74]. Another study compared 1421 metabolic genes in 1646 cancer samples obtained from 22 different tumors with 962 metabolic genes from normal tissues, finding minor differences in the expression levels between normal and cancer cells [72]. Finally, a more detailed analysis allowed Hu et al. [75] to add a few genes involved in glycolysis, nucleotide metabolism, or one-carbon pathway to the short list of genes upregulated in cancer. 

Intraoperative 13C-glucose infusion in a few non-small cell lung cancers has compared “in vivo” metabolism between tumors and healthy lungs. The results confirm the activation of glycolysis and add evidence for the oxidation of several nutrients, especially lactate, with indications of regional metabolic heterogeneity [76]. Additionally, an “in vivo” isotope tracing in human clear cell renal cell carcinoma indicates enhanced glycolysis and reduction in glucose oxidation as compared to adjacent normal kidneys [77]. Spatial metabolomics has been developed with success to reveal differences between subtypes of non-small-cell lung cancers: adenocarcinoma and squamous cell carcinoma. These are useful results since their therapy regimes are different [78].

In summary, according to the kind of analysis that has been performed, one may conclude that, between normal and cancer cells, one may observe a large variation of metabolism or, instead, a quite small one, according to the kind of analysis performed and the interpretation criteria adopted.

Functional analysis of about 50 pathways of intracellular metabolism in about 180 cancer cell lines, executed by clustering techniques, shows convergence towards two main metabolic pathways: glycolysis/hexosamine pathway on one side and TCA cycle/fatty acid biosynthesis on the other [79]. 

Several analyses focused on the effects of TME on cancer cells’ metabolism indicate that TME may modulate the supply of oxygen and availability of nutrients, including lactate, which affects metabolic pathways in cancer cells [80,81,82,83,84]. In particular, lactate may be converted to pyruvate by lactate dehydrogenase B and hence enter into the metabolism of cancer cells. On the other hand, lactate dehydrogenase A is engaged in the production and secretion of lactate from pyruvate [85]. Finally, TME has been found to interfere with cancer immunotherapy by forming a barrier to the attack from effector T cells [86]. Not only that, the microenvironment (hypoxia, acidosis, low glucose, osmotic pressure) of early cancer cells may promote the insurgence of Warburg phenotype and tumor aggressiveness [87,88]. 

The cancer metabolic rewiring, presented in Figure 2, allows us to propose a comprehensive interpretation for several reported findings, in which various, apparently scattered metabolic reactions, when targeted by specific drugs, strongly inhibit the proliferation of cancer cells. Let us make a few examples of these sensitive targets: ACLY [50,89,90,91,92,93]; mitochondrial shuttles [94,95]; transaminases [96,97,98]; one-carbon/serine pathway [99,100,101]; malonyl decarboxylase, the first enzyme of fatty acids biosynthesis [102]; enolase-1 [103]; dihydro-orotate dehydrogenase, in pyrimidine biosynthesis [104]. They are all relevant steps of growth metabolism (Figure 2). It does not escape our attention that in Figure 2, ACLY has a control position for the production of building blocks for cell proliferation. Finally, one has to recall that inhibition of metabolic targets is generally followed by metabolic rewiring, which may recover the ability to grow in cancer cells [73,74,75,76,77,78,79,80,81,82,83,84,85,86,87,88,89,90,91,92,93,94,95,96,97,98,99,100,101,102,103,104,105], eventually utilizing reactions of secondary metabolism.

## 5. Conclusions

For many years, and with fluctuating interest, the Warburg effect has been investigated without reaching an understanding of why fast-growing cancer cells have a respiro-fermentative metabolism. Only recently, at least a coherent reason to study this effect has been proposed: the need to open new ways for anticancer drug discovery to overcome the limitations still present in cancer treatment developed after adopting oncogene-targeting drugs and immunotherapy. In fact, Vander Heiden et al. [106] recognized that “a better understanding of mechanistic links between cellular metabolism and growth control may ultimately lead to better treatments for human cancer”.

Herein, it is presented, for the first time, a mechanistic network that accounts for the Warburg effect. It is based on the presence of two mitochondrial functionalities, one canonical and the other whose enzymatic endowment is restricted to a downsized set of TCA enzymes, in reciprocal redox balance, able to facilitate the production of building blocks for growth. These mitochondrial modifications are generated using a strongly stimulated glycolysis, characteristic of the Warburg effect, to inactivate OXPHOS pathway activity, either by glycolytic intermediates and/or by hypoxia [26,42], thereby provoking the formation of downsized mitochondrial activities. Hence, the Warburg (and Crabtree) effects are generated as “emergent properties” from the integration of a large number of metabolic reactions, bringing these studies into the field of systems biology [107], thereby opening the possibility to construct dynamic mathematical models able to analyze the role of each molecular component in generating the “emergent property” [108]. The requirement of this new paradigm may explain why reductionism, prevalent in past studies [109,110], has not been able, so far, to solve the riddle of the Warburg effect. 

A systems biology approach [31], planned with the aim to identify new anticancer drug targets, may be expected to activate the following main tasks: (1) multi-omics and imaging analysis in many different cancer cell lines and in tumors with or without the presence of known anticancer drugs, to investigate, for each condition, both the heterogeneity of mitochondrial enzymes endowment, as well to identify which metabolic pathways are executed in central carbon metabolism and in biosynthetic pathways; (2) develop constraint-based and dynamic mathematical models, utilizing data sets obtained for the various cancer types previously investigated. Simulation analyses should allow the extraction of the governing principles of cancer cell metabolism so as to offer a new rational basis for drug discovery. 

This new metabolism-driven systems approach will offer a shift of paradigm [111] to the growing field of precision oncology [112], which presently relies on statistical correlations obtained from large sets of heterogeneous data [113,114,115]. 

In conclusion, the working hypothesis presented in this review appears able to rationalize the connections of the Warburg effect with the overall growth metabolism of cancer cells and their unrestricted ability to proliferate, as foreseen by [106]. 

## Data Availability

No new original research data have been presented in this review.
